# Digestibility, Growth Performance, Body Measurement and Hormone of Sheep Fed with Different Levels of *Brachiaria decumbens* Diets

**DOI:** 10.21315/tlsr2023.34.1.5

**Published:** 2023-03-31

**Authors:** Mimi Syazwani Jaapar, Eric Lim Teik Chung, Nazri Nayan, Kalai Vaani Muniandy, Muhammad Hazziq Mohd Hamdan, Shokri Jusoh, Faez Firdaus Abdullah Jesse

**Affiliations:** 1 Department of Animal Science, Faculty of Agriculture, Universiti Putra Malaysia, 43400 UPM Serdang, Selangor, Malaysia; 2 Institute of Tropical Agriculture and Food Security, Universiti Putra Malaysia, 43400 UPM Serdang, Selangor, Malaysia; 3 Department of Veterinary Clinical Studies, Faculty of Veterinary Medicine, Universiti Putra Malaysia, 43400 UPM Serdang, Selangor, Malaysia

**Keywords:** Saponins, Nutrient Apparent Digestibility, Feed Efficiency, Body Index, Growth Hormone, Dorper, Saponin, Kebolehcernaan Ketara Nutrien, Kecekapan Makanan, Indeks Badan, Hormon Pertumbuhan, Dorper

## Abstract

Limited data are available regarding the effects of *Brachiaria decumbens* on sheep’s growth performance at different times. Therefore, this current study focused on sheep’s nutrient apparent digestibility, feed efficiency, body index, and growth hormone when they are fed with low and high levels of *B. decumbens* diets. A total of 30 six-month-old male Dorper cross sheep were divided randomly into three treatment groups with 10 sheep per treatment. Treatment 1 (control) sheep were fed with *Pennisetum purpureum* and pellets as the basal diet, whereas Treatment 2 and 3 sheep were fed with feed mixed with low (10%) and high (60%) levels of *B. decumbens*, respectively. The study was conducted in two phases consisting of short-term feeding (seven days) and long-term feeding (90 days). Throughout the experiment, daily fecal voided were collected in the morning for seven days continuous before the end of each feeding phases for the determination of nutrient apparent digestibility. The amount of feed offered and refusals plus body weight gain were recorded daily to determine the feed efficiency (FE). Besides, the body measurements of each sheep from every treatment were measured weekly and blood samples were collected for the analysis of growth hormone (GH) concentration. There were significant differences (*p* < 0.05) in the nutrient apparent digestibility, growth performance, body measurement, and GH concentration among treatment sheep throughout the study period. Treatment 3 sheep fed with 60% of *B. decumbens* diet revealed the lowest dry matter (DM), crude protein (CP), neutral detergent fiber (NDF), and acid detergent fiber (ADF) digestibility during the long-term feeding. Likewise, Treatment 3 (T3) sheep had the lowest total bodyweight gain, average daily gain, total feed intake, and daily feed intake among treatment sheep. The heart girth index (HGI) of T3 sheep was also significantly lower during the short-term feeding. Moreover, the GH concentration of T3 sheep was significantly lower as compared to the control that decreases steadily throughout the study period. In conclusion, high levels of *B. decumbens* showed the most significant results out of all three treatments indicating the presence of saponins, which produce negative effects on the sheep’s overall performance.

## Abstract (fr)

Data tersedia mengenai kesan jumlah *Brachiaria decumbens* yang berbeza terhadap prestasi pertumbuhan biri-biri pada tempoh masa yang berbeza adalah terhad. Oleh itu, kajian semasa ini memberi tumpuan kepada kebolehcernaan ketara nutrien, kecekapan makanan, indeks badan, dan hormon pertumbuhan bebiri yang diberi makan dengan diet *B. decumbens* pada tahap rendah dan tinggi. Sebanyak 30 ekor bebiri Dorper jantan berumur 6 bulan dibahagikan secara rawak kepada 3 kumpulan rawatan dengan 10 ekor bebiri setiap kumpulan. Rawatan 1 (kawalan) bebiri diberi makan dengan *Pennisetum purpureum* dan pelet sebagai diet asas, manakala bebiri Rawatan 2 dan 3 masing-masing diberi makan dengan campuran *B. decumbens* pada tahap rendah (10%) dan tinggi (60%). Kajian ini dijalankan dalam dua fasa yang terdiri daripada pemakanan jangka pendek (7 hari) dan panjang (90 hari). Sepanjang eksperimen, najis harian yang dibuang dikumpulkan pada waktu pagi selama 7 hari berterusan sebelum tamat setiap fasa pemakanan untuk menentukan kebolehcernaan ketara nutrien. Jumlah makanan yang diberi, sisa makanan, dan penambahan berat badan direkodkan setiap hari untuk menentukan kecekapan makanan (FE). Selain itu, ukuran badan setiap bebiri daripada setiap rawatan diukur setiap minggu serta sampel darah diambil untuk analisis kepekatan hormon pertumbuhan (GH). Terdapat perbezaan yang signifikan (*p* < 0.05) dalam kebolehcernaan ketara nutrien, prestasi pertumbuhan, ukuran badan, dan kepekatan GH di kalangan bebiri rawatan sepanjang tempoh kajian. Bebiri rawatan 3 (T3) yang diberi makan dengan 60% diet *B. decumbens* menunjukkan kebolehcernaan bahan kering (DM), protein kasar (CP), gentian detergen neutral (NDF), dan gentian detergen asid (ADF) yang paling rendah semasa pemberian makanan jangka panjang. Bebiri T3 mempunyai jumlah penambahan berat badan, purata kenaikan harian, jumlah pengambilan makanan, dan pengambilan makanan harian yang paling rendah di kalangan bebiri rawatan. Indeks lilitan jantung (HGI) bebiri T3 juga jauh lebih rendah semasa pemberian makanan jangka pendek. Selain itu, kepekatan GH bebiri T3 adalah jauh lebih rendah berbanding dengan bebiri kawalan yang menurun secara berterusan sepanjang tempoh kajian. Kesimpulannya, tahap *B. decumbens* yang tinggi menunjukkan keputusan yang paling ketara daripada ketiga rawatan yang menunjukkan kehadiran saponins boleh menghasilkan kesan negatif terhadap prestasi keseluruhan bebiri.

**Highlights**
- Different levels of *Brachiaria decumbens* in diets were proven to affect the production performance of sheep.
- Sheep fed with 60% of *B. decumbens* diet demonstrated the worst nutrient digestibility, growth performance, body measurement and growth hormone among treatment sheep.
- Sheep fed with 10% of *B. decumbens* diet showed no to minimal effects as compared to the control sheep throughout the study period.

## INTRODUCTION

*Brachiaria* spp. is a perennial grass originated from East Africa and there are seven species available, which include *B. decumbens, B. brizantha, B. arrecta, B. dictyoneura, B. humidicola, B. mutica* and *B. ruziziensis* (Low 2015). *Brachiaria* spp. have been planted on more than 80% of total improved pasture land with *B. decumbens* as the most favoured species. This species could provide all the forage requirements of ruminants in the tropics and this has helped to enhance the production performance of livestock (Chung *et al*. 2018). *B. decumbens* is a low-growing, trailing, perennial grass with upright, sword-shaped leaves and the grass can spread by both rhizomes and stolon as well as through seed production (Assumaidaee & Mustapha 2012). Furthermore, it exhibits tolerance to low soil fertility (Rosa *et al*. 2016), drought resistance (Faccin *et al*. 2014), and is relatively free from pests and diseases (Abdullah & Rajion 1997).

On the other hand, *B. decumbens* has the potential to increase live weight gain in ruminants owing to its high nutritive value such as dry matter digestibility and crude protein that are important for animal growth (Low 2015). For instance, *B. decumbens* that was defoliated every five weeks for two years, were found to contain higher dry matter and crude protein contents, compared to *B. brizantha* and *B. ruziziensis* (Vendramini *et al*. 2014). According to Aregheore (2001), the nitrogen content of *B. decumbens* ranges from 10 g to 28 g N/kg dry matter with 56 to 78% digestibility within five weeks range of grass regrowth period. Nevertheless, the livestock production on this pasture system has been variable even when the quality and quantity of the pasture are high (Low 2015; Jaapar *et al*. 2022). There have been many reports of sporadic outbreaks of photosensitivity, general ill-thrift, and mortality in livestock due to the naturally occurring toxic compound found in *B. decumbens* (Muniandy *et al*. 2020). These potentially toxic compounds have been isolated from leaf and stem fractions of *B. decumbens*, which is identified as steroidal saponins associated with secondary hepatogenous photosensitization in ruminants (Gomar *et al*. 2005).

From the plant’s viewpoint, saponins are related to the defense mechanism by acting as a protective chemical barrier, protecting themselves against predator, insects, and by having adverse effects on herbivores (Cardona-Alvarez *et al*. 2016). Sheep are more susceptible to *B. decumbens* poisoning than other ruminants, and sheep under one year of age are more susceptible to toxicity poisoning than adults, which can occur at any time of year and at any maturity stage of the plant (Riet-Correa *et al*. 2011; Faccin *et al*. 2014). Clinical symptoms such as liver damage, hepatic jaundice, and photosensitization have been observed in sheep fed with *B. decumbens* as the main source of feed, indicating hepatotoxicity (Assumaidaee *et al*. 2010; Muniandy, Chung, Reduan, *et al*. 2021). Consequently, the present study aims to determine the effects of low and high levels of *B. decumbens* diets on the nutrient apparent digestibility, feed efficiency, body measurement, as well as growth hormone concentration of sheep during both short- and long-term feeding.

## MATERIALS AND METHODS

### Saponins Extraction and Measurement

A total of 10, 20, 30, 40, 50, 60, 70, 80, 90 and 100% of *B. decumbens* were mixed with *Pennisetum purpureum* and underwent saponin extraction and measurement according to Yuliana *et al*. (2014) to determine the low and high levels of *B. decumbens*. The mixed diets were oven-dried at 50°C for 12 h and ground to pass 0.5 mm sieve. Then, 10 mL of each 100% methanol was added to a test tube containing 0.5 g of the sample then placed in an ultrasonic water bath (Barnstead/Lab Line Aqua Wave 9377, E60H, Germany) for extraction for 20 min at room temperature. Each sample was centrifuged (Thermo Scientific IEC Centra CL2 Centrifuge, Fisher Scientific Pte Ltd, Singapore) at 4°C for 10 min and repeated twice. The samples were then calibrated against Diosgenin standard (Sigma-Aldrich D1634, Sigma Aldrich Chemie GmbH, Steinheim, Germany). The samples were added with 0.2 mL vanillin, 0.25 mL ethanol and 2.5 mL 72% H_2_SO_4_, and vortexed. The samples were heated in a water bath (Watson Victor Ltd., Bw6t, Watson Victor Limited, New Zealand) at 60°C for 10 min. After cooled, the absorbance was read in UV-Vis spectrophotometer (UV-Vis spectrophotometer, U-1800, 5930482, High Technology Corporation, Tokyo, Japan) at a wavelength of 544 nm. The standard curve linear regression equation was calculated using the standard and the sample’s saponin concentration was tabulated. 10% of *B. decumbens* mixture was selected as the low level, while 60% of *B. decumbens* mixture was selected as the high level because the level of saponins was peaked at 60% and maintained high throughout the 70, 80, 90 and 100% of *B. decumbens* mixture. Meanwhile, 100% *P. purpureum* was used as the basal diet and served as control. The nutrient compositions of each feed are presented in Table 1.

### Animals

The experimental protocols and ethics were approved by the Institutional Animal Care and Use Committee (Approval number: UPM/IACUC/AUP-R046/2019). A total of 30 six-month-old male Dorper cross sheep were purchased and randomly divided into three treatment groups consisted of 10 sheep each. Treatment 1 (T1) control sheep were fed with *Pennisetum purpureum* and pellet as the basal diet, whereas treatments 2 (T2) and 3 (T3) sheep were fed with feed mixed with low (10%) and high (60%) levels of *B. decumbens*, respectively. Those sheep were dewormed orally once in the morning with Albendazole 2.5% and acclimatised for two weeks before the start of the experiment. All sheep were placed in an individual metabolic pen and were fed with cut grass and supplemented with pellets at the rate of 70:30 ration/animal/day. The amount of feed given was calculated based on the bodyweight needed by each sheep and the amount increased weekly. Water was available *ad libitum*.

### Nutrient Apparent Digestibility

Daily fecal voided were collected in the morning, weighed, and 10% (w/w) of the fecal were stored frozen at −20°C for the first seven days during the short-term feeding, while the same steps were repeated during the last seven consecutive days of feeding trial during the long-term feeding for the determination of nutrient apparent digestibility. At the end of the collection period, the fecal samples of each sheep were pooled and subsampled for proximate analysis. Diets and fecal samples were analysed for dry matter (DM), organic matter (OM), crude protein (CP), ether extract (EE), neutral detergent fiber (NDF), and acid detergent fiber (ADF). The proximate composition of the diets and fecal samples were determined following the method of the Association of Official Analytical Chemists (AOAC, 1995), while the NDF, and ADF were determined using Van Soest *et al*. (1991) technique. The apparent digestibility was determined by this equation:


Apparent digestibility (%)=[(Nutrient in feed-Nutrient in feces)/Nutrient in feed]×100

### Growth Performance

Throughout the study, the feed refusal and feed intake (FI) were weighed and recorded daily by measuring the difference between the feed offered and refusal. The body weight of each sheep was also recorded weekly prior to morning feeding using the standard weight tape. The feed efficiency (FE) for each lamb was calculated from the total FI and total weight gained during the study period.

### Body Measurement

The body measurements of each sheep from every treatment were measured weekly as described by Mavule *et al*. (2013). The body length (BL), heart girth (HG), and whither height (WH) was measured to the nearest (cm) after restraining and holding lambs in an unforced position throughout the 90 days trial before feeding. The relative growth of lambs, the body length index (BLI), heart girth index (HGI), and somatic index (SI) were then calculated using these equations:


$$\begin{array}{l} \text{BLI}=\text{BL}/\text{WH};\\ \text{HGI}=\text{HG}/\text{WH};\;\text{and}\\ \text{SI}=\text{HG}/\text{BL.} \end{array}$$

### Growth Hormone Analysis

On day 0, 7, 30, 60 and 90, blood samples were collected via jugular venipuncture (5 mL into Serum BD Vacutainer® blood collection tube) for serum collection before morning feeding. The blood collected was centrifuged at 3000 round per minute (rpm) for 5 min to obtain the serum and stored at −80°C before growth hormone analysis. The concentrations of growth hormone (GH) in blood serum samples were measured using an enzyme-linked immunosorbent assay (ELISA) kit according to the manufacturer’s protocol (BT-Laboratory, Shanghai) with specification standards for GH. The assay had a sensitivity of 0.045 ng/mL with the detection range between 0.1 ng/mL and 40 ng/mL.

### Statistical Analysis

Based on the output parameter of the G*power analysis, the number of animals used was sufficient to obtain an actual power of 0.8. All data collected were analysed using Statistical Analysis Software version 9.4 (SPSS). Shapiro-Wilk test was used to check for normality of data where *p* > 0.05 was considered as normally distributed data. Then, the data were further subjected to the one-way analysis of variance (ANOVA) to determine the effects of *B. decumbens* levels on nutrient digestibility, growth performance, body measurement, and growth hormone concentration. Tukey Kramer (Honest Significant Difference) post-hoc test was used to compare the mean differences among groups at a 5% level of significance. The data were considered significant at *p* < 0.05.

## RESULTS

### Nutrient Apparent Digestibility

The nutrient apparent digestibility of sheep fed with low and high levels of *B. decumbens* diets during the short- and long-term feeding is illustrated in Table 2. No significant differences (*p* > 0.05) were observed in all the nutrient digestibility parameters except for the DM digestibility during the short-term feeding. Significantly lower (*p* < 0.05) dry matter digestibility was exhibited in T3 sheep in contrast to T1 sheep.

During the long-term feeding, significant differences (*p* < 0.05) were observed in the DM, CP, NDF, and ADF among treatment sheep. There were no significant differences (*p* > 0.05) in the organic matter and metabolised energy among treatment sheep. Comparing to the control sheep, T3 sheep fed with 60% of *B. decumbens* diet revealed the lowest DM, CP, NDF and ADF digestibility proposing an impaired nutrient digestibility along the gastrointestinal tract, which will ultimately affect the growth performance of sheep.

### Growth Performance

Table 3 shows the growth performance of sheep fed with low and high levels of *B. decumbens* diets during the short- and long-term feeding. There were no significant differences (*p* > 0.05) in all the parameters among treatments during the short-term feeding. Numerically, T3 sheep demonstrated lower growth performance parameters leading to the higher feed efficiency followed by T2 as compared to T1 sheep on the first seven days of the study period.

On the other hand, there were significant differences (*p* < 0.05) in the total bodyweight gain, average daily gain, total feed intake, and daily feed intake among treatment sheep during the long-term feeding. As compared to the control, T3 sheep exhibited the lowest total bodyweight gain, average daily gain, total feed intake, and daily feed intake indicating the poorest growth performance. Likewise, T3 sheep also had the lowest final bodyweight and feed efficiency numerically despite not having significant differences.

Overall, T3 sheep fed with the highest *B. decumbens* diet signifying the poorest growth performance followed by T2 sheep. Meanwhile, T1 sheep not fed with any *B. decumbens* diet had the best growth performance throughout the 90 days study period.

### Body Measurement

Table 4 present the body measurement and body index of sheep fed with low and high levels of *B. decumbens* diets during the short- and long-term feeding. There were no significant differences (*p* > 0.05) in all the body measurement parameters for all treatment groups.

For body indexes, only heart girth index during the short-term feeding showed significant difference (*p* < 0.05). However, the value of indexes decreases as the *B. decumbens* level increases. Despite no significant differences, T3 sheep fed with 60% of *B. decumbens* demonstrated a smaller body size numerically with a lower body length index, heart girth index, and somatic index at the end of the study period as compared to the control sheep suggesting a stunted growth.

### Growth Hormone Concentration

The growth hormone concentration of sheep fed with different levels of *B. decumbens* diets throughout the 90 days feeding trial is depicted in Table 5. No significant difference (*p* > 0.05) was observed in the GH concentration at day 0 among treatment sheep.

Significant differences (*p* < 0.05) of the GH concentration were detected on day 7, 30, 60, and 90 among treatment sheep. As opposed to the control sheep, both *B. decumbens* treatments revealed a significantly lower GH concentration that decreases steadily throughout the study period.

The GH concentration of T2 sheep decreased by 27.40%, whereas T3 sheep revealed the highest reduction of GH concentration by 33.48% on day 90 indicative of a suppressed growth hormone secretion. This will then affect the growth performance of sheep as reported earlier.

## DISCUSSION

### Nutrient Digestibility

Favourable growth performance and productivity of sheep were highly associated with the high efficiency of optimum ruminal fermentation, nutrient digestibility and effective nutrient utilisation (Zheng *et al*. 2021). In the present study, high *B. decumbens* level in the feed demonstrated decreased nutrients digestibility of DM, CP, NDF and ADF. Agreeing to Wina *et al*. (2005), the decrease in nutrients digestibility might be attributed to the lower fibrolytic enzyme activity in the rumen due to the presence of anti-nutritional factors or steroidal saponins in *B. decumbens*. The forestomach is the first metabolic region, as saponins are hydrolyzed in the rumen, omasum, and abomasum into sapogenins (epsimilagenin, episarsapogenin, smilagenone and smilagenin) before being absorbed in the duodenum and jejunum, which will then be transported to the liver via transportal vein (Muniandy *et al*. 2020). According to a study conducted by Johnson *et al*. (1986), the active mucosal transport was inhibited in the presence of saponins. With the saponins level surpassing sublethal, there were possibilities of implications for the uptake of macromolecules from passing through the epithelium of the intestinal mucosal (Gee *et al*. 1996). Consequently, the nutrient absorption capacity required by the sheep was significantly decreased in exchange for the entrance of macromolecules, which supports the result of the current study. For example, Potter *et al*. (1993) reported that saponins are able to reduce the digestibility of saponin-protein complexes. The decrease in nutrient digestibility and feed intake due to the high *B. decumbens* level could be the main reason for the reduced CP digestibility of sheep in the present study, thus leading to poor growth performance. The result was consistent with the reports by Hess *et al*. (2004) and Santoso *et al*. (2007), where the CP, OM, and NDF loss in feces increased with the presence of *Sapindus Saponaria*, *Sapindus mukrossi* and *Biophytum petersianum* containing high amounts of saponins. In addition, the digestibility of fibrous fraction tends to decrease with slower digestion due to the presence of steroidal saponins, thus require a longer time to undergo the breakdown process into smaller particles appropriate for fermentation and absorption in the rumen (Granzotto *et al*. 2011). As a result, the lower levels of DM, NDF and ADF digestibility reported in this study were attributed to the effects of saponins on rumen fermentation and microbiota.

### Growth Performance

Despite the fact *B. decumbens* is one of the most cultivated species in South America, Asia, and the South Pacific region, they are not used as the main grass for feeding ruminants due to the concern related to the toxicity of the steroidal saponins (Chung *et al*. 2018). T3 sheep fed with the highest *B. decumbens* diet recorded the poorest growth performance as compared to T2 and control sheep. The declined growth performance and feed efficiency could be due to factors such as feed palatability, feed avoidance and toxin effects. These events might be attributed to the fact that *B. decumbens* containing saponins are naturally occurring surface-active glycosides, which have a bitter and astringent taste for animals (Cheok *et al*. 2014). The low growth performances produced by the sheep in this study may arise from poor feed palatability leading to poor feed intake. Aazami *et al*. (2013) mentioned that avoidance behavior of ruminants could also justify poor growth rates and weight loss that were often associated with livestock grazing on *B. decumbens* pasture. Specifically, avoidance of ingesting this pasture will indirectly decrease nutrient intake and thus, reduce growth rates. Supporting the idea, this study observed that T3 sheep fed with higher levels of *B. decumbens* tend to have greater feed leftover. This finding corroborates a study on saponin-containing plants (Alghirani *et al*. 2021), where a decrease in feed intake was observed in animals fed diet supplemented with Yucca extract containing saponins. On the other hand, risk of toxicity has become a limiting factor when *B. decumbens* is fed as the main source of feed (Chung *et al*. 2018). One of the clinical signs of *B. decumbens* toxicity is secondary or hepatogenous photosensitisation. *B. decumbens* intoxication causing liver dysfunction leads to an elevation in the concentration of liver enzymes (Muniandy Chung, Jaapar, *et al*. 2021), alteration in liver metabolism, thereby resulting in weight loss in 70% of studied animals (Lelis *et al*. 2018). Sheep are more susceptible to *B. decumbens* poisoning than other ruminants, and sheep under one year of age are more susceptible to toxicity poisoning than adults (Riet-Correa *et al*. 2011). This corroborates the findings from the current study as T3 sheep fed with the highest level of *B. decumbens* demonstrated the poorest growth performance and decreased digestibility most probably due to some degree of intoxication.

### Body Measurement

Body measurements could be useful in defining the performance traits of animals, especially in growing animals (Cam *et al*. 2010). In this study, despite having no significant differences, the value for each body measurement variable and body index did reduce as the level of *B. decumbens* increases. According to a previous study, saponins administration had no significant effect on body measurements with inconsistent results among treatment groups (Aazami *et al*. 2013). Nonetheless, Liu *et al*. (2018) obtained a significant difference in the HGI value at day 60 to 90, whereas the rest of the body measurements showed no significant differences. Similarly, to this study, only HGI during the short-term feeding showed significant difference. The disparities in the results might be due to the age of the sheep during the feeding trial. According to Salako (2006), young sheep had relatively higher correlation coefficients in body measurements that can be measured in younger sheep compared to adult sheep. This is because younger animals will undergo predominant metamorphic growth at this stage, which caused some of the body parameters to increase significantly than other stages of production. Besides, sheep showed greater susceptibility to *B. decumbens* toxicity compared to cattle and goats with most cases affecting young animals (Muniandy *et al*. 2020), hence affecting the body measurement. The other effect of *B. decumbens* toxicity is the presence of birefringent crystals that are capable of blocking the bile ducts, provides concrete links between the saponins in *B. decumbens* and rumen metabolism, and the absorption of the derived compound (Miles *et al*. 1993). Macrophages were also observed surrounding the crystal along with damaged liver, which later affect the kidneys during the chronic phase. This demonstrates that the dysfunctional internal organs led to degradation of growth performance, which was reflected in the body measurement. In brief, there are very few studies on the effect of saponins or saponin-containing plants on body measurements, thus no final revelation of this parameter was obtained, indicating more future studies need to be conducted.

### Growth Hormone Concentration

Growth potential, modification in body composition, as well as average daily gain in animals are indicated by both insulin-like growth factor-1 and GH (Abdel-Raheem & Hassan 2021). The secretion of GH by the pituitary gland is controlled by at least two hypothalamic factors, which are stimulatory GH-releasing factor and inhibitory factor somatostatin (SRIH). There is increasing evidence that nutritional status plays a major role in determining GH concentrations, particularly in ruminants. In the present study, significant differences were observed between treatments throughout the 90-day feeding trial as the GH concentration decreases in response to the increasing level of *B. decumbens* level in diets. Nonetheless, this finding contradicts the reports from all previous studies. For instance, both Zhong *et al*. (2012) and Liu *et al*. (2018) reported that no significant effect was detected on the levels of plasma hormones (GH) in lambs fed with *Astragulus membranaceus* and tea leaves containing saponins, respectively. Conversely, higher plasma GH concentrations were obtained from underfed (low protein) lactating cows (Hart *et al*. 1978). In line with less feed intake, the inflation of GH might be linked to the utilisation of the hormone to mobilise energy and exchange of metabolites from adipose tissue to lean tissue for metabolism as the dietary nutrient was inadequate. Hart *et al*. (1985) proved further that the GH response to growth hormone-releasing factor of sheep was also improved during restricted feeding as opposed to *ad libitum* feeding, insinuating that the reduction in feed intake was the possible reason for the elevated growth hormone due to poorly supplied nutrition. Based on previous studies, all the results reported were inconsistent with the findings obtained in the present study. This could be governed by the time of exposure, as well as the toxin effect or steroidal saponins presence in *B. decumbens*. However, this study is the first attempt to relate the effect of feeding *B. decumbens* on the plasma GH concentration. Therefore, the effect of plant secondary metabolites on the plasma hormones of ruminants remains controversial, which warrants further investigation.

## CONCLUSION

In summary, different levels of *B. decumbens* diets were proven to affect the growth performance, growth hormone concentration, body measurement, and nutrient apparent digestibility of sheep at different time phases. T2 sheep displayed minimal effects in this *in vivo* assessment as only a low level of *B. decumbens* was distributed to the sheep. Meanwhile, T3 showed the most significant results out of all three treatments indicating the presence of saponins, which did produce negative effects on the sheep’s overall performance. Based on the results obtained from the present study, farmers are highly advised to avoid using fresh *B. decumbens* as their main source of feed for sheep owing to the detrimental effects of steroidal saponins present in the grass.

## Figures and Tables

**Table 1:** Nutritional composition of T1, T2 and T3 diets.

Composition	T1 (Control)	T2	T3
DM % as fed	24.12 ± 0.83^a^	19.12 ± 0.20^b^	17.38 ± 0.46^b^
OM	95.63 ± 0.29^a^	95.24 ± 0.14^ab^	94.90 ± 0.01^b^
CP	16.92 ± 0.12^a^	16.41 ± 0.13^a^	15.03 ± 0.15^b^
EE	2.22 ± 0.11^a^	1.92 ± 0.10^ab^	1.71 ±0.03^b^
NDF	63.64 ± 0.19^a^	60.71 ± 1.04^a^	54.16 ± 0.90^b^
ADF	42.37 ± 1.48^a^	38.53 ± 0.959^b^	36.15 ± 0.43^b^
ADL	5.23 ± 0.25^a^	4.54 ± 0.24^ab^	3.69 ± 0.29^b^
GE (kJ/kg DM)	15.73 ± 0.20^a^	16.37 ± 0.32^a^	16.69 ± 0.56^a^

*Note*: T1: 100% *P. purpureum*, T2: 10% of *B. decumbens* + 90% *P. purpureum*, T3: 60% of *B. decumbens* + 40% of *P. purpureum*. DM: Dry matter, OM: Organic matter, CP: Crude protein, EE: Ether extract, NDF: Neutral detergent fiber, ADF: Acid detergent fiber, ADL: Acid detergent lignin, GE: Gross Energy. All values were expressed as mean ± SE;

a,b values with superscript within row are significantly different at *p* < 0.05.

**Table 2:** Nutrient apparent digestibility of sheep fed with low and high levels of *B. decumbens* diets during the short- and long-term feeding.

Parameter	T1 (control)	T2	T3
Day 7 (short term feeding)			

DM (%)	62.95 ± 2.28^a^	60.71 ± 2.33^ab^	54.56 ± 2.52^b^
OM (%)	65.55 ± 3.02	61.73 ± 2.69	56.87 ± 2.48
CP (%)	61.86 ± 3.15	58.35 ± 2.14	54.57 ± 1.88
NDF (%)	58.92 ± 3.90	54.89 ± 2.00	52.15 ± 2.15
ADF (%)	48.85 ± 1.90	46.33 ± 2.70	43.17 ± 2.03
ME (kJ/kg DM)	8.40 ± 0.17	8.05 ± 0.17	8.00 ± 0.19

Day 90 (long term feeding)			

DM (%)	63.73 ± 1.91^a^	59.02 ± 3.01^ab^	55.48 ± 2.33^b^
OM (%)	65.17 ± 2.11	62.22 ± 3.52	56.77 ± 2.50
CP (%)	60.74 ± 1.18^a^	56.41 ± 1.23^b^	53.24 ± 1.29^b^
NDF (%)	59.55 ± 0.95^a^	53.97 ± 1.96^ab^	52.18 ± 2.15^b^
ADF (%)	49.70 ± 2.14^a^	47.82 ± 1.55^ab^	42.84 ± 2.12^b^
ME (kJ/kg DM)	8.58 ± 0.21	8.26 ± 0.22	7.92 ± 0.16

*Note*: All values were expressed as mean ± SE;

a,b values with superscript within row are significantly different at *p* < 0.05.

T1: 100% *P. purpureum*, T2: 10% of *B. decumbens* + 90% *P. purpureum*, T3: 60% of *B. decumbens* + 40% of *P. purpureum*. DM: Dry matter; OM: Organic matter; CP: Crude protein; NDF Neutral detergent fiber; ADF: Acid detergent fiber; ME: Metabolised energy.

**Table 3:** Growth performance of sheep fed with low and high levels of *B. decumbens* diets during the short- and long-term feeding.

Parameter	T1 (Control)	T2	T3
Day 7 (short term feeding)			

Initial bodyweight (kg)	17.23 ± 1.06	17.18 ± 0.57	16.86 ± 0.63
Final bodyweight (kg)	17.88 ± 1.00	17.80 ± 0.59	17.45 ± 0.61
Total bodyweight gain (kg)	0.77 ± 0.11	0.62 ± 0.11	0.60 ± 0.04
Average daily gain (g/d)	0.67 ± 0.95	0.51 ± 0.51	0.52 ± 0.72
Total feed intake (kg DMI)	1.83 ± 0.10	1.48 ± 0.13	1.21 ± 0.07
Daily feed intake (kg DMI/d)	0.26 ± 0.18	0.21 ± 0.27	0.17 ± 0.18
Feed efficiency (kg DMI/kg gain)	2.06 ± 0.22	2.69 ± 0.57	3.45 ± 0.59

Day 90 (long term feeding)			

Final bodyweight (kg)	24.97 ± 0.74	23.73 ± 0.95	23.43 ± 1.04
Total bodyweight gain (kg)	7.96 ± 0.20^a^	7.03 ± 1.70^ab^	6.30 ± 0.60^b^
Average daily gain (g/d)	2.37 ± 0.13^a^	2.00 ± 0.11^ab^	1.60 ± 0.10^b^
Total feed intake (kg DMI)	30.39 ± 0.99^a^	28.53 ± 1.76^ab^	28.35 ± 2.73^b^
Daily feed intake (kg DMI/d)	0.41 ± 0.13^a^	0.39 ± 0.13^ab^	0.38 ± 0.11^b^
Feed efficiency (kg DMI/kg gain)	3.81 ± 0.06	4.07 ± 0.71	4.68 ± 0.84

*Notes*: All values were expressed as mean ± SE;

a,b values with superscript within row are significantly different at *p* < 0.05.

T1: 100% *P. purpureum*, T2: 10% of *B. decumbens* + 90% *P. purpureum*, T3: 60% of *B. decumbens* + 40% of *P. purpureum*.

**Table 4:** Body measurement and body index of sheep fed with low and high levels of *B. decumbens* diets during the short- and long-term feeding.

Parameter	T1 (Control)	T2	T3
Day 7 (short term feeding)			

Body measurement:			
Whither Height (cm)	52.66 ± 0.88	53.00 ± 0.93	50.00 ± 2.45
Body Length (cm)	45.33 ± 1.12	44.66 ± 1.26	44.50 ± 1.09
Neck Length (cm)	16.66 ± 0.33	16.50 ± 0.50	15.83 ± 0.31
Neck Circumference (cm)	24.33 ± 0.33	24.00 ± 0.63	23.66 ± 0.56
Heart Girth Circumference (cm)	60.00 ± 3.90	58.33 ± 1.28	57.66 ± 0.88
Abdominal Circumference (cm)	77.83 ± 2.29	76.16 ± 1.56	76.16 ± 1.33
Right Forelimb (cm)	46.50 ± 1.23	45.66 ± 1.23	45.00 ± 0.68
Left Forelimb (cm)	46.00 ± 0.89	45.66 ± 1.23	45.16 ± 0.95
Right Hindlimb (cm)	49.83 ± 0.60	49.83 ± 1.01	48.50 ± 0.76
Left Hindlimb (cm)	50.33 ± 0.84	49.83 ± 0.79	48.83 ± 0.79
Body index:			
BLI	0.98 ± 0.18	0.86 ± 0.03	0.84 ± 0.03
HGI	1.41 ± 0.14^a^	1.11 ± 0.31^b^	1.09 ± 0.31^b^
SI	1.37 ± 0.19	1.29 ± 0.04	1.30 ± 0.02

Day 90 (long term feeding)			

Body measurement:			
Whither Height (cm)	56.66 ± 0.88	55.66 ± 0.88	54.66 ± 0.88
Body Length (cm)	47.66 ± 1.33	47.33 ± 1.33	47.33 ± 1.33
Neck Length (cm)	18.00 ± 0.33	18.00 ± 0.57	17.66 ± 0.33
Neck Circumference (cm)	24.66 ± 0.67	24.33 ± 0.67	24.00 ± 0.58
Heart Girth Circumference (cm)	63.00 ± 0.58	60.33 ± 0.88	57.83 ±0.87
Abdominal Circumference (cm)	80.66 ± 2.84	82.33 ± 3.84	78.33 ± 2.84
Right Forelimb (cm)	49.33 ± 0.66	47.66 ± 1.21	46.33 ± 0.67
Left Forelimb (cm)	49.33 ± 0.67	48.00 ± 1.52	46.33 ± 0.67
Right Hindlimb (cm)	52.00 ± 0.57	51.66 ± 1.76	50.66 ± 1.76
Left Hindlimb (cm)	52.66 ± 0.88	50.66 ± 1.76	50.33 ± 1.76
Body index:			
BLI	0.87 ± 0.02	0.86 ± 0.04	0.84 ± 0.34
HGI	1.10 ± 0.02	1.10 ± 0.01	1.02 ± 0.05
SI	1.28 ± 0.02	1.29 ± 0.06	1.22 ± 0.02

*Notes*: All values were expressed as mean ± SE;

a,b values with superscript within row are significantly different at *p* < 0.05.

T1: 100% *P. purpureum*, T2: 10% of *B. decumbens* + 90% *P. purpureum*, T3: 60% of *B. decumbens* + 40% of *P. purpureum*. BLI: Body length index, HGI: Heart girth index, SI: Somatic index.

**Table 5:** Growth hormone concentration (ng/mL) of sheep fed with low and high levels of *B. decumbens* diets throughout 90 days.

Day	T1 (Control)	T2	T3
0	7.10 ± 0.12^a^	6.97 ± 0.14^a^	6.75 ± 0.22^a^
7	6.75 ± 0.17^a^	5.47 ± 0.12^b^	5.72 ± 0.34^b^
30	6.99 ± 0.09^a^	5.47 ± 0.18^b^	4.16 ± 0.12^c^
60	6.83 ± 0.15^a^	4.88 ± 0.19^b^	4.12 ± 0.39^b^
90	6.99 ± 0.11^a^	5.06 ± 0.24^b^	4.49 ± 0.23^c^

*Notes*: All values were expressed as mean ± SE;

a,b,c values with superscript within row are significantly different at *p* < 0.05.

T1: 100% *P. purpureum*, T2: 10% of *B. decumbens* + 90% *P. purpureum*, T3: 60% of *B. decumbens* + 40% of *P. purpureum*.

## References

[b1-tlsr-34-1-67] Aazami MH, Tahmasbi AM, Ghaffari MH, Naserian AA, Valizadeh R, Ghaffari AH (2013). Effects of saponins on rumen fermentation, nutrients digestibility, performance, and plasma metabolites in sheep and goat kids. Annual Research & Review in Biology.

[b2-tlsr-34-1-67] Abdel-Raheem SM, Hassan EH (2021). Effects of dietary inclusion of *Moringa oleifera* leaf meal on nutrient digestibility, rumen fermentation, ruminal enzyme activities and growth performance of buffalo calves. Saudi Journal of Biological Sciences.

[b3-tlsr-34-1-67] Abdullah AS, Rajion MA (1997). Dietary factors affecting entero-hepatic function of ruminants in the tropics. Animal Feed Science and Technology.

[b4-tlsr-34-1-67] Alghirani MM, Chung ELT, Sabri DSM, Tahir MNJM, Kassim NA, Kamalludin MH, Nayan N, Jesse FFA, Sazali AQ, Loh TC (2021). Can *Yucca schidigera* be used to enhance the growth performance, nutrient digestibility, gut histomorphology, cecal microflora, carcass characteristic, and meat quality of commercial broilers raised under tropical conditions?. Animals.

[b5-tlsr-34-1-67] Aregheore EM (2001). Nutritive value and utilization of three grass species by crossbred Anglo-Nubian goats in Samoa. Asian-Australasian Journal of Animal Sciences.

[b6-tlsr-34-1-67] Association of Official Analytical Chemists (AOAC) (1995). Official methods of analysis.

[b7-tlsr-34-1-67] Assumaidaee AJM, Mustapha NM (2012). Toxicity of signal grass (*Brachiaria decumbens*): A review article. Journal of Advances in Medicine and Medical Research.

[b8-tlsr-34-1-67] Assumaidaee AA, Saad MZ, Sabri J, Noordin MM (2010). The role of oxidative stress in Brachiaria decumbens toxicity in sheep. Pertanika Journal of Tropical Agricultural Science.

[b9-tlsr-34-1-67] Cam MA, Olfaz M, Soydan E (2010). Body measurements reflect body weights and carcass yields in Karayaka sheep. Asian Journal of Animal and Veterinary Advances.

[b10-tlsr-34-1-67] Cardona-Álvarez J, Vargas-Vilória M, Paredes-Herbach E (2016). Clinical and histopathological study of the phototoxic dermatitis in Zebu calves in grazing of *Brachiaria decumbens*. Revista MVZ Cordoba.

[b11-tlsr-34-1-67] Cheok CY, Salman HAK, Sulaiman R (2014). Extraction and quantification of saponins: A review. Food Research International.

[b12-tlsr-34-1-67] Chung ELT, Predith M, Nobilly F, Samsudin AA, Jesse FFA, Loh TC (2018). Can treatment of *Brachiaria decumbens* (signal grass) improve its utilisation in the diet in small ruminants? A review. Tropical Animal Health and Production.

[b13-tlsr-34-1-67] Faccin TC, Riet-Correa F, Rodrigues FS, Santos AC, Melo GK, Silva JA, Ferreira R, Itavo CCBF, Lemos RA (2014). Poisoning by *Brachiaria brizantha* in flocks of naïve and experienced sheep. Toxicon.

[b14-tlsr-34-1-67] Gee JM, Wortley GM, Johnson IT, Price KR, Rutten AA, Houben GF, Penninks AH (1996). Effects of saponins and glycoalkaloids on the permeability and viability of mammalian intestinal cells and on the integrity of tissue preparations in vitro. Toxicology in Vitro.

[b15-tlsr-34-1-67] Gomar MS, Driemeier D, Colodel EM, Gimeno EJ (2005). Lectin histochemistry of foam cells in tissues of cattle grazing *Brachiaria spp*. Journal of Veterinary Medicine Series A.

[b16-tlsr-34-1-67] Granzotto F, Branco AF, dos Santos AL, Barreto JC, Teixeira S, Serrano RC, Barbosa OR, Mano DS, Ferelli F, Coneglian SM (2011). Proteic supplements with and without sulfur sources on intake behavior of steers fed with low quality hay. Semina: Ciencias Agrarias.

[b17-tlsr-34-1-67] Hart IC, Bines JA, Moran SV, Ridley JL (1978). Endocrine control of energy metabolism in the cow: comparison of the levels of hormones (prolactin, growth hormone, insulin and thyroxine) and metabolites in the plasma of high-and low-yielding cattle at various stages of lactation. Journal of Endocrinology.

[b18-tlsr-34-1-67] Hart IC, Chadwick PME, James S, Simmonds AD (1985). Effect of intravenous bovine growth hormone or human pancreatic growth hormone-releasing factor on milk production and plasma hormones and metabolites in sheep. Journal of Endocrinology.

[b19-tlsr-34-1-67] Hess HD, Beuret RA, Lotscher M, Hindrichsen IK (2004). Ruminal fermentation, methanogenesis and nitrogen utilization of sheep receiving tropical grass hay-concentrate diets offered with *Sapindus saponaria* fruits and *Cratylia argentea* foliage. Journal of Animal Science.

[b20-tlsr-34-1-67] Jaapar MS, Chung ELT, Nayan N, Kamalludin MH, Saminathan M, Muniandy KV, Hamdan MHM, Jusoh S, Jesse FFA (2022). Effects of different levels of *Brachiaria decumbens* diets on *in vitro* gas production and ruminal fermentation. Journal of Animal Health and Production.

[b21-tlsr-34-1-67] Johnson IT, Gee JM, Price K, Curl C, Fenwick GR (1986). Influence of saponins on gut permeability and active nutrient transport in vitro. The Journal of Nutrition.

[b22-tlsr-34-1-67] Lelis DL, Rennó LN, Chizzotti ML, Pereira CER, Silva JCP, Moreira LGT, Carvalho FBP, Chizzotti FHM (2018). Photosensitization in naïve sheep grazing signal grass (*Brachiaria decumbens*) under full sunlight or a silvopastoral system. Small Ruminant Research.

[b23-tlsr-34-1-67] Liu C, Qu YH, Guo PT, Xu CC, Ma Y, Luo HL (2018). Effects of dietary supplementation with alfalfa (*Medicago sativa* L.) saponins on lamb growth performance, nutrient digestibility, and plasma parameters. Animal Feed Science and Technology.

[b24-tlsr-34-1-67] Low SG (2015). Signal grass (*Bracharia decumbens*) toxicity in grazing ruminants. Agriculture.

[b25-tlsr-34-1-67] Mavule BS, Muchenje V, Bezuidenhout CC, Kunene NW (2013). Morphological structure of Zulu sheep based on principal component analysis of body measurements. Small Ruminant Research.

[b26-tlsr-34-1-67] Miles CO, Wilkins AL, Munday SC, Flayen A, Holland PT, Smith BL (1993). Identification of insoluble salts of the beta-D-glucuronides of episarsasapogenin and epismilagenin in the bile of lambs with alveld and examination of *Narthecium ossifragum*, *Tribulus terrestris*, and *Panicum miliaceum* for sapogenins. Journal of Agricultural and Food Chemistry.

[b27-tlsr-34-1-67] Muniandy KV, Chung ELT, Jaapar MS, Hamdan MHM, Reduan MFH, Salleh A, Jesse FFA (2021). The influence of feeding low and high level of *Brachiaria decumbens* diets on the hematology, serum biochemistry, and acute phase proteins of sheep. Tropical Animal Health and Production.

[b28-tlsr-34-1-67] Muniandy KV, Chung ELT, Jaapar MS, Hamdan MHM, Salleh A, Jesse FFA (2020). Filling the gap of *Brachiaria decumbens* (signal grass) research on clinico-pathology and haemato-biochemistry in small ruminants: A review. Toxicon.

[b29-tlsr-34-1-67] Muniandy KV, Chung ELT, Reduan MFH, Paul BT, Jaapar MS, Hamdan MHM, Jesse FFA (2021). Clinico-pathological responses of sheep to graded levels of *Brachiaria decumbens* diets. Journal of Advanced Veterinary Research.

[b30-tlsr-34-1-67] Potter SM, Jimenez-Flores R, Pollack J, Lone TA, Berber-Jimenez MD (1993). Protein saponin interaction and its influence on blood lipids. Journal of Agricultural and Food Chemistry.

[b31-tlsr-34-1-67] Riet-Correa B, Castro MB, Lemos RA, Riet-Correa G, Mustafa V, Riet-Correa F (2011). *Brachiaria spp*. poisoning of ruminants in Brazil. Pesquisa Veterinaria Brasileira.

[b32-tlsr-34-1-67] Rosa FB, Rubin MI, Martins TB, de Lemos RA, Gomes DC, Pupin RC, Lima SC, Barros CS (2016). Spontaneous poisoning by *Brachiaria decumbens* in goats. Pesquisa Veterinaria Brasileira.

[b33-tlsr-34-1-67] Salako AE (2006). Application of morphological indices in the assessment of type and function in sheep. International Journal of Morphology.

[b34-tlsr-34-1-67] Santoso B, Kilmaskossub A, Sambodo P (2007). Effects of saponins from *Biophytum petersianum Klotzsch* on ruminal fermentation, microbial protein synthesis and nitrogen utilization in goats. Animal Feed Science and Technology.

[b35-tlsr-34-1-67] Van Soest PJ, Robertson JB, Lewis BA (1991). Methods for dietary fiber, neutral detergent fiber, and nonstarch polysaccharides in relation to animal nutrition. Journal of Dairy Science.

[b36-tlsr-34-1-67] Vendramini JMB, Sollenberger LE, Soares AB, Silva WL, Sanchez JMD, Valente AL, Aguiar AD, Mullenix MK (2014). Harvest frequency affects herbage accumulation and nutritive value of brachiaria grass hybrids in Florida. Tropical Grasslands-Forrajes Tropicales.

[b37-tlsr-34-1-67] Wina E, Muetzel S, Becker K (2005). The impact of saponins or saponin-containing plant materials on ruminant production: A review. Journal of Agricultural and Food Chemistry.

[b38-tlsr-34-1-67] Yuliana P, Laconi EB, Wina E, Jayanegara A (2014). Extraction of tannins and saponins from plant sources and their effects on in vitro methanogenesis and rumen fermentation. Journal of the Indonesian Tropical Animal Agriculture.

[b39-tlsr-34-1-67] Zheng C, Zhou J, Zeng Y, Liu T (2021). Effects of mannan oligosaccharides on growth performance, nutrient digestibility, ruminal fermentation and hematological parameters in sheep. PeerJ.

[b40-tlsr-34-1-67] Zhong RZ, Yu M, Liu HW, Sun HX, Cao Y, Zhou DW (2012). Effects of dietary *Astragalus polysaccharide* and *Astragalus membranaceus* root supplementation on growth performance, rumen fermentation, immune responses, and antioxidant status of lambs. Animal Feed Science and Technology.

